# A conserved predicted pseudoknot in the NS2A-encoding sequence of West Nile and Japanese encephalitis flaviviruses suggests NS1' may derive from ribosomal frameshifting

**DOI:** 10.1186/1743-422X-6-14

**Published:** 2009-02-05

**Authors:** Andrew E Firth, John F Atkins

**Affiliations:** 1 BioSciences Institute, University College Cork, Cork, Ireland; 2 Department of Human Genetics, University of Utah, Salt Lake City, UT 84112-5330, USA

## Abstract

Japanese encephalitis, West Nile, Usutu and Murray Valley encephalitis viruses form a tight subgroup within the larger *Flavivirus *genus. These viruses utilize a single-polyprotein expression strategy, resulting in ~10 mature proteins. Plotting the conservation at synonymous sites along the polyprotein coding sequence reveals strong conservation peaks at the very 5' end of the coding sequence, and also at the 5' end of the sequence encoding the NS2A protein. Such peaks are generally indicative of functionally important non-coding sequence elements. The second peak corresponds to a predicted stable pseudoknot structure whose biological importance is supported by compensatory mutations that preserve the structure. The pseudoknot is preceded by a conserved slippery heptanucleotide (Y CCU UUU), thus forming a classical stimulatory motif for -1 ribosomal frameshifting. We hypothesize, therefore, that the functional importance of the pseudoknot is to stimulate a portion of ribosomes to shift -1 nt into a short (45 codon), conserved, overlapping open reading frame, termed *foo*. Since cleavage at the NS1-NS2A boundary is known to require synthesis of NS2A in *cis*, the resulting transframe fusion protein is predicted to be NS1-NS2A^N-term^-FOO. We hypothesize that this may explain the origin of the previously identified NS1 'extension' protein in JEV-group flaviviruses, known as NS1'.

## Findings

The genus *Flavivirus *(see [1-3] for reviews) includes species such as Dengue virus, Japanese encephalitis virus (JEV), West Nile virus (WNV), Tick-borne encephalitis virus and Yellow fever virus. Also within the family *Flaviviridae *are Hepatitis C, Hepatitis G and the *Pestivirus *genus. The Japanese encephalitis *group *includes JEV, WNV, Murray Valley encephalitis virus (MVEV), Usutu virus and St Louis encephalitis virus (SLEV). These important human pathogens are transmitted by mosquitoes and can cause potentially fatal encephalitis. The single-stranded positive sense genomic RNA is ~11 kb in length and contains a single long open reading frame, translated as a polyprotein that is cleaved by virus-encoded and host proteases to produce ~10 mature proteins.

Inspired by the 'suppression of synonymous site variation' (SSSV) statistic of ref. [4], we decided to investigate conservation at synonymous sites in the *Flaviviridae *family. For a given species or group within the family, the polyprotein coding sequences (CDSs) were extracted, translated, aligned with CLUSTALW [5], and back-translated to nucleotide sequence alignments. Beginning with pairwise sequence comparisons, conservation at synonymous sites (only) was evaluated by comparing the observed number of base substitutions with the number expected under a neutral evolution model. The procedure takes into account whether synonymous site codons are 1-, 2-, 3-, 4- or 6-fold degenerate and the differing probabilities of transitions and transversions (full details are available on request from the authors). Statistics were then summed over a phylogenetic tree as described in [6], and averaged over a sliding window.

**Figure 1:**
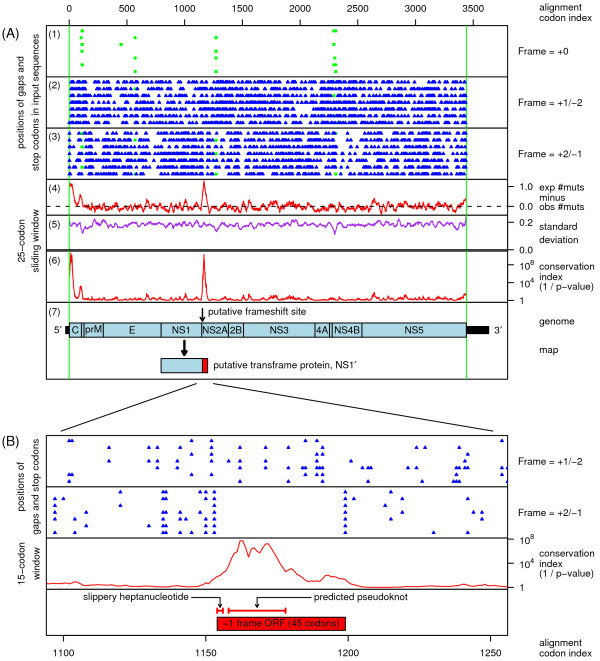
**Conservation at synonymous sites in Japanese encephalitis and related viruses**. Conservation at synonymous sites was calculated for an input alignment comprising the polyprotein CDSs from the seven JEV-group sequences listed in the caption to Figure 2. **(A) **Panels 1–3 show the positions of stop codons (blue triangles) in the three forward reading frames. The +0 frame is the polyprotein frame and is therefore devoid of stop codons. Alignment gaps are indicated in green. Panel 4 shows the difference between the expected number (assuming neutral evolution) and observed number of base substitutions at synonymous sites, summed over a phylogenetic tree, and averaged over a 25-codon sliding window. Panel 5 shows the estimated standard deviation for the statistic in panel 4 – major dips tend to correspond to alignment gaps (fewer pairwise sequence comparisons to *sum *over), while the rise at each end of the alignment corresponds to the shorter terminal windows over which statistics are *averaged*. Panel 6 is an approximation of the *p*-value corresponding to the statistic in panel 4, albeit subject to the assumption of normal distribution. Panel 7 shows a map of the JEV genome, indicating the position of the putative -1 ribosomal frameshift site, and the putative transframe protein which may equate to NS1'. **(B) **Zoom-in of the region corresponding to the conservation peak in the NS2A CDS with a 15-codon sliding window. Note the highest level of conservation corresponds precisely to the region covered by the predicted pseudoknot.

When this procedure was applied to the JEV group (excepting SLEV; see below), two striking peaks in synonymous site conservation were found – one within the capsid CDS (very 5' end of the polyprotein CDS) and one at the 5' end of the NS2A CDS (Figure 1A). The peak within the capsid CDS is a common feature of flavivirus genomes, with this sequence region playing important roles in replication and, for some species, translation initiation (reviewed in [1]). On the other hand, the peak within the NS2A CDS was either not present, or not so pronounced, outside of the JEV group. One unexplored possiblity is that this second highly conserved region plays a role in packaging. However, based on the particular features of the sequence in this region, and on relevant previously published data, we hypothesize an alternative role.

**Figure 2:**
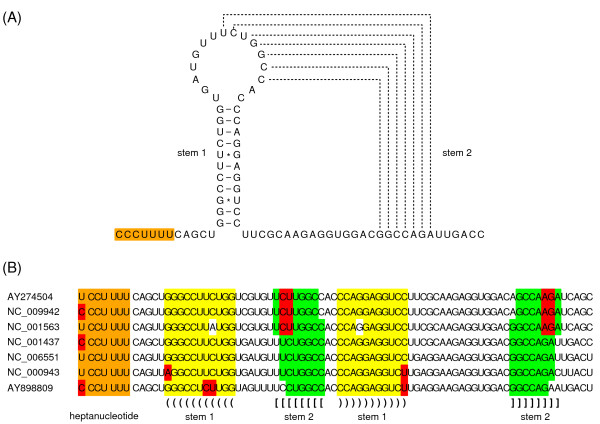
**Putative stimulatory elements for ribosomal -1 frameshifting**. **(A) **The slippery heptanucleotide and predicted 3' RNA pseudoknot structure for JEV [GenBank:NC_001437]. **(B) **Sequence alignment of different JEV-group sequences, showing the conserved presence of a slippery heptanucleotide (Y CCU UUU; orange) and potential to form a 3' RNA pseudoknot. Base-pairings in stem 1 are indicated with '()'s and yellow background while base-pairings in stem 2 are indicated with '[]'s and green background. Base substitutions that preserve base-pairings or the slippery heptanucleotide are highlighted in red. Viruses: Japanese encephalitis (JEV) – [GenBank:NC_001437]; West Nile (WNV; lineage I) – [GenBank:NC_009942]; West Nile (WNV; lineage II) – [GenBank:NC_001563]; Kunjin – [GenBank:AY274504]; Murray Valley encephalitis (MVEV) – [GenBank:NC_000943]; Alfuy – [GenBank:AY898809]; Usutu – [GenBank:NC_006551].

Many viruses harbour sequences that induce a portion of ribosomes to shift -1 nt and continue translating in the new reading frame [7]. The -1 frameshift site typically consists of a 'slippery' heptanucleotide fitting the consensus motif N NNW WWH, where NNN are any three identical nucleotides, WWW represents AAA or UUU, H represents A, C or U, and spaces separate zero-frame codons. This is followed by a 'spacer' region of 5–9 nt, and then a stable RNA secondary structure such as a pseudoknot or hairpin. Inspection of the conserved sequence at the 5' end of the NS2A CDS in the JEV group revealed the potential to form a GC-rich stable pseudoknot structure (Figure 2) in the region precisely corresponding to the peak in synonymous site conservation (Figure 1B). The predicted pseudoknot is well-supported by a number of compensatory mutations, including three separate instances of an A:U pair being replaced by a G:C pair. Furthermore, where stem 1 is destabilized in one sequence by a G:A mispairing, stem 2 is lengthened by an extra base-pairing. Positioned 5' of the pseudoknot, and separated from it by a 5 nt spacer, is a conserved Y CCU UUU heptanucleotide, where Y represents C or U, and spaces separate polyprotein-frame codons. Allowing for G:U anticodon:codon repairing at position 1 of the heptanucleotide (when Y = U), the combination of the Y CCU UUU heptanucleotide and the 3' pseudoknot fit the consensus motif for -1 frameshifting.

The -1 frame ORF (termed *foo*, for "Flavivirus Overlapping ORF") comprises 45 codons. Such short out-of-frame ORFs are not well-represented amongst known cases of programmed ribosomal frameshifting, but this may be more a consequence of the difficulty in finding such cases rather than any inherent rarity. Indeed we recently demonstrated the occurrence of -1 frameshifting, at a level of 10–18%, into a short ORF overlapping the 6K CDS in the *Alphavirus *genus [8].

The combination of slippery heptanucleotide, 3' pseudoknot, and 45-codon -1 frame ORF is conserved in all five RefSeqs (listed in the caption to Figure 2), and essentially all their GenBank 'genome neighbours' [9] as of December 2008 (223 sequences). The only exceptions are seven sequences – four with single mispairings in stem 1 of the pseudoknot, one with a shortened stem 2, and two with a truncated -1 frame ORF. At least three of the seven are annotated as attenuated.

The putative shift site is located at codons 8–9 of the NS2A CDS. Thus frameshifting would result in a 52 amino acid NS2A^N^-FOO fusion peptide (where NS2A^N ^represents the N-terminal nine amino acids of NS2A). Previous work has demonstrated that, at least in Dengue virus, cleavage at the non-standard NS1-NS2A cleavage site requires translation of substantial (≫ 9 amino acids) parts of NS2A [10-12]. Thus NS2A^N^-FOO is likely not cleaved from NS1, i.e. the predicted mature transframe protein is NS1-NS2A^N^-FOO.

On protein gels, NS1 typically migrates as a cluster of bands, partly due to differing degrees of glycoslyation which can add ~6 kDa to the NS1 mass [13-15]. NS1 also forms multiple disulfide bonds and migrates with a substantially lower apparent molecular mass under non-reducing conditions [16]. In JEV, MVEV and WNV, however, the existance of an elongated form of NS1, termed NS1', has been demonstrated [13-15,17-19]. NS1' is N-terminally coincident with NS1 [15] but extends into NS2A, as demonstrated by the presence of an epitope not present in NS1 but present in polyprotein sequence overlapping the carboxy terminus of NS1 [15,17,18], and by the necessity of NS2A coding sequence for NS1' expression [15]. Thus, NS1' has been proposed to result from cleavage at an alternative site within NS2A. However, sites proposed by ref. [20] are too far downstream to account for NS1' [15], and attempts to localize the cleavage site by determining the carboxy terminal sequence of NS1' have been unsuccessful [15]. Pulse-chase experiments have demonstrated that NS1' is not simply a precursor of NS1 but is, instead, a stable end product [13,15].

**Figure 3:**
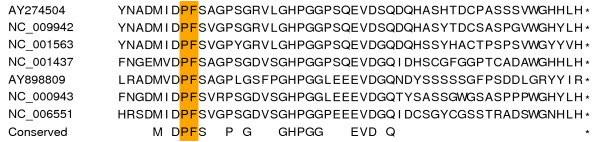
**Peptide sequences for the putative transframe extension of NS1**. Predicted peptide sequences for NS2A^N^-FOO – the putative transframe extension of NS1 – for various JEV-group viruses. The shift-site amino acids are indicated in the orange rectangle, and the *foo *termination codon is represented by an '*'. See caption to Figure 2 for virus names.

We hypothesize that, in JEV-group viruses, NS1' may in fact correspond to NS1-NS2A^N^-FOO. This putative product contains sufficient NS2A sequence to potentially provide the polyprotein-derived epitope not in NS1. Furthermore, although early estimates of the mass difference between NS1 and NS1' are inconsistent with the hypothesis (e.g. unglycosylated masses of 42 kDa and 52 kDa, respectively, in JEV [13]), more recent estimates put the mass difference in a plausible range (7–8 kDa) for the mass of NS2A^N^-FOO (5.3–5.7 kDa), especially if it is post-translationally modified, or migrating more slowly than expected due to its high proline content (4–6 prolines; Figure 3).

For example, working with JEV, ref. [14] estimated masses for glycosylated NS1 and NS1' of 48 kDa and 55 kDa, respectively, while for unglycosylated NS1 and NS1' the masses were 42 kDa and 49 kDa. Thus, for JEV, the mass of the C-terminal extension in NS1' is ~7 kDa. Similarly, working with MVEV, ref. [15] estimated masses of 45 kDa and 53 kDa for glycosylated NS1 and NS1', and 39 kDa and 47 kDa for unglycosylated NS1 and NS1'. Thus, for MVEV, the mass of the C-terminal extension in NS1' is ~8 kDa. Similar results were obtained by ref. [18].

Consistent with our hypothesis, when ref. [21] expressed what they supposed to be an approximatation of JEV NS1' from a plasmid containing NS1 and the first 60 amino acids of NS2A (hereafter NS1-NS2A^1..60^), they appeared to obtain an NS1' doublet – consistent with a mixture of the zero-frame (NS1-NS2A^1..60^) and the predicted transframe (NS1-NS2A^N^-FOO) products. No such doublet was observed in controls comprising lysates from JEV-infected cells. Assuming the product that comigrates with wild-type NS1' is NS1-NS2A^N^-FOO, then the other product corresponds to a fainter, more rapidly migrating band. This is plausible if the high proline content of FOO causes it to migrate more slowly and if the artificial product NS1-NS2A^1..60 ^is more rapidly degraded. The data do not fit a cleavage hypothesis (as then the uncleaved NS1-NS2A^1..60 ^would be expected to migrate more *slowly *than wild-type NS1') and an impaired glycosylation explanation seems unlikely (since the doublet appears to be present even in a sample treated with endoglycosidase F). No NS1 was produced from the NS1-NS2A^1..60 ^plasmid (consistent with NS1-NS2A cleavage requiring synthesis of NS2A; see above) and no NS1' was produced from a plasmid just expressing NS1 (consistent with NS1' requiring the 5' end of the NS2A CDS).

A corresponding frameshift stimulatory motif was not found in SLEV – the most divergent of the six JEV-group RefSeqs but, interestingly, there was a long (89–165 codons) -1 frame ORF overlapping the boundary between NS1 and NS2A, so it is possible that frameshifting also occurs in SLEV at a non-canonical site, possibly further 5' such as within the NS1 CDS. No evidence for frameshifting was found in Dengue or Kokobera viruses – consistent with the apparent absence of NS1' in these species [19]. In contrast to JEV-group NS1', the elongated NS1 product (NS1-2A*; [22]) seen in Yellow fever virus apparently results from cleavage much closer to the carboxy terminus of NS2A [22,23] and, in any case, NS1-2A* appears to be simply a precursor of mature NS1 rather than a stable end product in itself, as demonstrated by pulse-chase analyses [22].

## Competing interests

The authors declare that they have no competing interests.

## Authors' contributions

AEF carried out the bioinformatic analyses and wrote the manuscript. Both authors edited and approved the final manuscript.
